# Supplementary material for: Design, Synthesis, and Biological Evaluation of N-Acylhydrazones and Their Activity Against Leishmania amazonensis Promastigotes
Source: Microorganisms. 2025 Jul 2;13(7):1563. doi: 10.3390/microorganisms13071563 (PMC12298860; doi:10.3390/microorganisms13071563)
Supplement: Supplementary file 1 [file microorganisms-13-01563-s001.zip › RMN amostras-20241114T155656Z-001.zip!/RMN amostras/composto 30 - PMM 03-18 13C.pdf]

PMM 03-18 - Joao Mafra
Op. Eliane

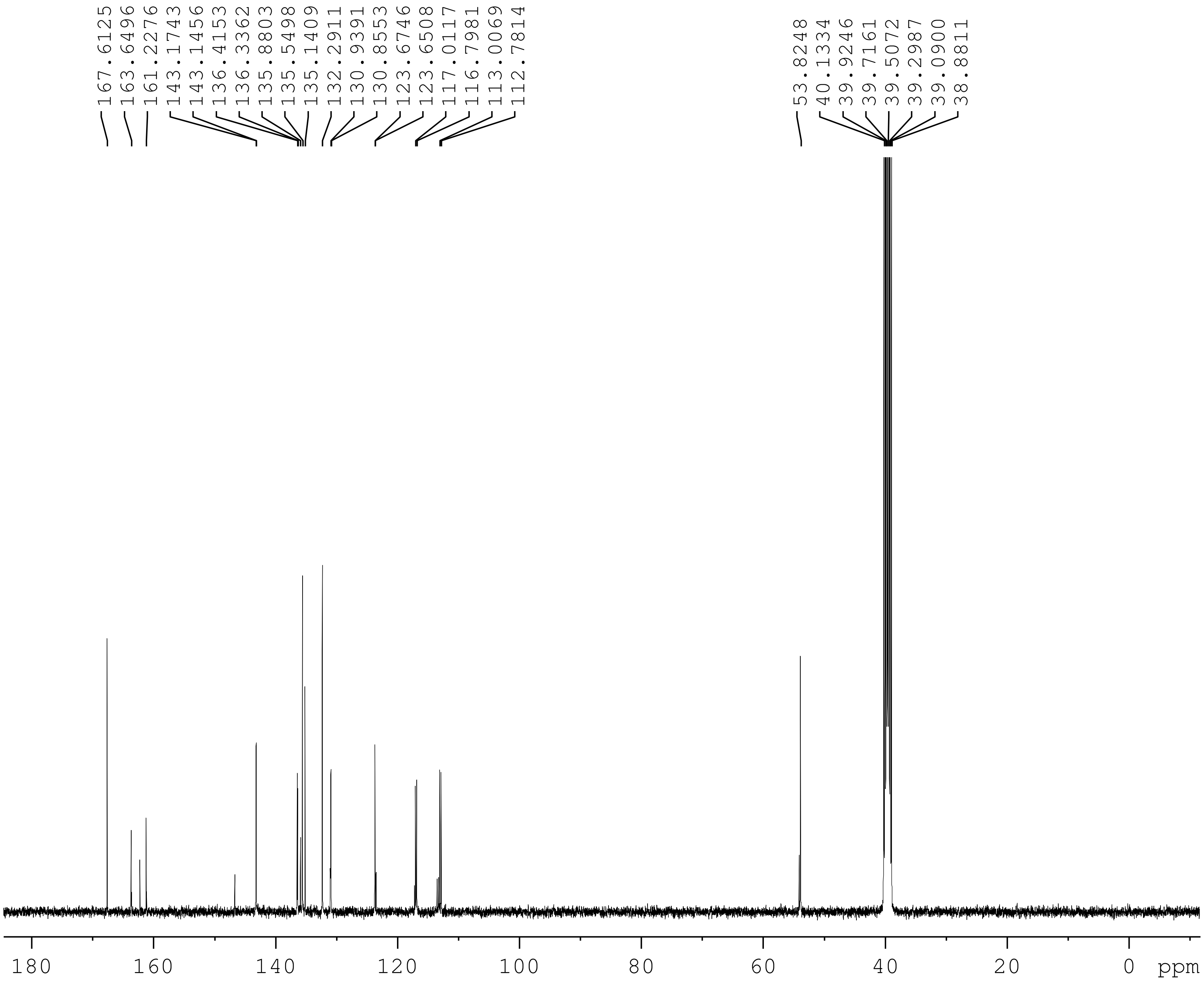

farmanguinhos
Instituto de Tecnologia em Fármacos

Current Data Parameters
NAME si107182302
EXPNO 11
PROCNO 1

F2 - Acquisition Parameters
Date_ 20180720
Time 15.39 h
INSTRUM spect
PROBHD Z122623_0041 (
PULPROG zgpg30
TD 65356
SOLVENT DMSO
NS 2048
DS 4
SWH 24038.461 Hz
FIDRES 0.735616 Hz
AQ 1.3594048 sec
RG 66.74
DW 20.800 usec
DE 18.00 usec
TE 298.0 K
D1 1.00000000 sec
D11 0.03000000 sec
TD0 1
SFO1 100.6278593 MHz
NUC1 13C
P1 10.00 usec
PLW1 35.50000000 W
SFO2 400.1516006 MHz
NUC2 1H
CPDPRG[2 waltz16
PCPD2 90.00 usec
PLW2 7.30000019 W
PLW12 0.11919000 W
PLW13 0.05995100 W

F2 - Processing parameters
SI 32768
SF 100.6178442 MHz
WDW EM
SSB 0
LB 1.00 Hz
GB 0
PC 1.40
